# Can Shared Mobility Compensate for Public Transport Disruptions? The Case of Milan’s Bike Sharing System During the COVID-19 Pandemic

**DOI:** 10.1177/03611981221123241

**Published:** 2022-09-20

**Authors:** Georgia Liouta, Giorgio Saibene, Niels van Oort, Oded Cats, Frederik Schulte

**Affiliations:** 1 Delft University of Technology, Delft, The Netherlands; 2 University of Hamburg, Hamburg, Germany

**Keywords:** pedestrians, bicycles, human factors, bike sharing, public transportation, capacity, elderly, Mobility as a Service, shared, transit, crowding

## Abstract

The COVID-19 pandemic poses an unprecedented challenge for public transport systems. The capacity of transport systems has been significantly reduced because of the social distancing measures. Therefore, new avenues to increase the resilience of public urban mobility need to be explored. In this work, we investigate the integration of bike sharing and public transport systems to compensate for limited public transport capacity under the disruptive impacts of the COVID-19 pandemic. As a first step, we develop a data analysis model to integrate the demand of the two underlying systems. Next, we build an optimization model for the design and operation of hybrid mixed-fleet bike sharing systems. We analyze the case of the subway and public bike sharing systems in Milan to assess this approach. We find that the bike sharing system (in its current state) can only compensate for a minor share of the public transport capacity, as the needs in fleet and station capacity are very high. However, the resilience of public urban mobility further increases when new design concepts for the bike sharing system are considered. An extension to a hybrid free-floating bike and docked e-bike system doubled the covered demand of the system. An extension of the station capacity of about 37% yields an additional increase of the covered demand by 6.5%–7.5%. On the other hand, such a hybrid mixed-fleet bike sharing system requires many stations and a relatively large fleet to provide the required mobility capacity, even at low demand requirements.

The global impact of the COVID-19 pandemic on the mobility and transportation sector has been established in multiple cases. Because of the high transmissibility of the virus, the outbreak was recognized as a pandemic in March 2020 (*1*). Measures such as quarantine, lockdown, social distancing, travel restrictions, closing of restaurants and schools, and isolation help to reduce the spread of corona viruses and are followed by many governments (*2*[Bibr bibr3-03611981221123241]–*4*). The proposed measures for social distancing in closed places have a great impact on the mobility capacity of public transport systems (PTSs) (*5*, *6*).

PTSs are mostly closed, crowded spaces that increase the chances of transmitting viruses such as the COVID-19 virus from infected to uninfected people (*7*, *8*). COVID-19-related studies found a significant impact of infections in public transit systems on the overall infection speed (*9*) as well as a relation between the number of new daily cases and the number of mobility trips performed in the preceding three weeks (*10*). The aforementioned studies conclude that PTSs are sources of COVID-19 virus transmission. Therefore, social distancing measures have been implemented that reduce the mobility capacity of PTSs. The result of the implementation of these measures can be seen, for instance, in the case of the Milan subway in Italy (illustrated in Figure 1).

**Figure 1.**
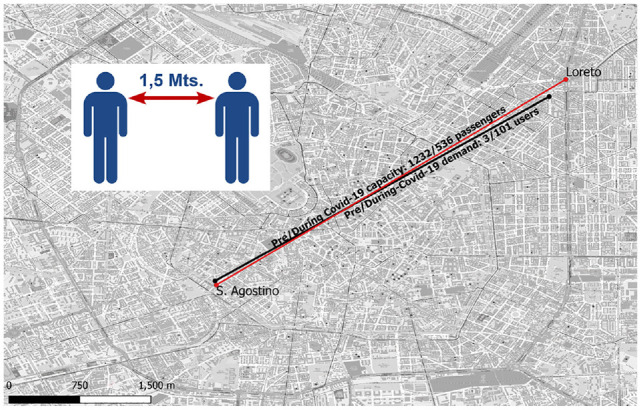
Social distancing measures affect the capacity of public transport systems. Here, it is shown how the implementation of the general rule of social distancing (i.e., 1.5 m between passengers) affects the capacity of subway trains and the bike sharing system (BSS) demand in Milan. The red dots represent subway stations, while the black dots represent BSS stations. (Color online only.)

The limited capacity of PTSs because of the COVID-19 distancing measures (i.e., mostly 1–2 m between passengers), the precautionary behavior of people, and the gradual return to normal life rhythms lead to PTSs being in unprecedented states (*11*, *12*). A central issue is the excessive demand, which is not satisfied by the PTSs and pushes for an alternative that enables people to move safely. There are several alternatives that can be integrated with PTSs to create systems that can accommodate this new situation. However, traffic congestion in most cities and air pollution are calling for green alternatives that do not burden the network too much. In line with this paradigm, the city of Milan has converted public roads into bicycle lanes during the pandemic. Furthermore, subsidies for personal bicycles have been discussed by several authors (*13*). In particular, subsidies for e-bikes are recognized as policy options during the pandemic. Personal bicycles may present an alternative whenever the PTS capacity is limited. However, in contrast to bicycles as part of a bike sharing system (BSS), these bicycles do not add to the publicly available mobility options. A recent study on public transit strikes (*14*) concludes that integrating BSSs into the PTS increases the system’s resilience to disruptive events. Moreover, several studies investigate the interplay of bike sharing and public transport in cities such as Poznań (*15*), Oslo (*16*), and Vienna (*17*). Recent literature has further stressed the importance of reliable service quality in shared mobility (*18*), possibly implemented in different service zones (*19*). Nevertheless, hardly any research considers disruptive events comparable to the COVID-19 pandemic and, to the best of our knowledge, there is no study available that explicitly considers the COVID-19 scenario. In this work, we propose and evaluate an operational integration of PTSs and BSSs to meet the mobility demand of cities in the face of disruptive events such as the recent COVID-19 pandemic. With this integration and the efficient design and operation of the BSS, a new integrated PTS is created that is suitable to deal with excessive unsatisfied demand because of the social distancing measures or similar capacity-limiting disruptive events.

The main challenge in implementing this integrated alternative is the method of designing and operating the BSS to provide safe mobility for all unsatisfied demand (i.e., demand exceeding the capacity implied by the 1.5 m distance criterion). Thus, the proposed approach is supply-oriented. In this work, we focus on the optimal design and operation of a mixed-fleet hybrid BSS (MFHBSS) considering the COVID-19 situation and aiming to create an integrated PTS. To this end, we propose a data analysis model to integrate the demand of the two underlying systems and develop an optimization model with a maximal location covering approach (*20*) for the design and operation of a MFHBSS. Such a system integrates free-floating and station-based bike sharing with electric and conventional bicycles to consider the requirements of elderly passengers. We analyze the case of the subway and public BSSs in Milan to evaluate the proposed methodology.

## Related Work

The reduced public transport capacity caused by social distancing measures, the increased likelihood of the virus spreading in PTSs, the need for people to keep moving, and reservations against PTSs strengthen the need to find a safe alternative to satisfy the mobility demand of people. The safe alternative, which could be integrated with current PTSs to maintain mobility capacity, could be BSSs. This choice is reinforced by many cities around the world, as they try to deal with social distances measures, becoming more friendly to pedestrians and cyclists by providing them with more urban space (*21*, *22*). Moreover, in this situation, there is a surge of people turning to BSSs (*23*, *24*), as bike sharing is perceived to be safer than public transit (*25*), increasing the importance of bike sharing as an alternative to public transit.

BSSs can complement or substitute existing PTSs (*17*, *26–28*). Moreover, a BSS can be a solution in the event of a long-term or short-term disruption of the PTS (*14*, *29*, *30*). An element to consider for the efficiency of a BSS is its design and operation. This type of problem can be addressed by optimization models. The main objective categories of these models are the maximization of demand coverage (*31*[Bibr bibr32-03611981221123241][Bibr bibr33-03611981221123241]–*34*), the minimization of transportation costs and overall costs (*35*[Bibr bibr36-03611981221123241][Bibr bibr37-03611981221123241]–*38*), and the maximization of profit (*39*, *40*). Moreover, there are studies that use different approaches to design and operate a BSS, such as simulation approaches (*41*[Bibr bibr42-03611981221123241]–*43*). Table 1 presents the above studies in summary.

**Table 1.** Summary Table of Past Research on BSS Design and Operation

Reference	Problem	Objective				Method	Case
		MDC	MUD	MP	MC		
Caggiani et al. (*35*)	Bike station	na	na	na	√	ILP	AC
Çelebi et al. (*31*)	Bike station	na	√	na	na	MINLP	Istanbul
Fernández et al. (*41*)	Bike location	na	na	na	na	ABS	Madrid
Frade and Ribeiro (*32*)	Bike station	na	na	na	na	na	na
	Bike relocation	√	na	na	na	LP	Coimbra
Jian et al. (*42*)	Bike allocation	na	na	na	na	na	na
	Dock allocation	na	√	na	na	SO	New York City
Lin and Yang (*36*)	Bike station	na	na	na	na	na	na
	Bikeways	na	na	na	√	INLP	AC
Martinez et al. (*39*)	(E)Bike station	na	na	na	na	na	na
	(E)Bike relocation	na	na	√	na	MILP	Lisbon
Park and Sohn (*33*)	Bike station	√	na	na	na	BILP	Seoul
Saharidis et al. (*34*)	Bike station	na	√	na	na	PILP	Athens
Sayarshad et al. (*40*)	Bike station	na	na	na	na	na	na
	Bike relocation	na	na	√	na	ILP	Tehran
Soriguera et al. (*43*)	Bike rebalancing	na	na	na	na	na	na
	Bike relocation	na	na	na	√	ABS	Barcelona
Yan et al. (*37*)	Bike station	na	na	na	na	na	na
	Bike relocation				√	MILP	Taipei
Yuan et al. (*38*)	Bike station,	na	na	na	na	na	na
	Bike relocation	na	na	na	√	MILP	Beijing
This paper	Bike station	na	na	na	na	na	na
	E-bike station	√	na	na	na	MILP	Milan
	(E)Bike relocation						

*Note*: MDC = maximization of demand coverage; MUD = minimization of unmet demand; MP = maximization of profit; MC = minimization of costs. LP = linear program; ILP = integer linear program; MILP = mixed-integer linear program; INLP = integer non-linear program; MINLP = mixed-integer non-linear program; BILP = binary integer linear program; PILP = pure integer linear program; SO = simulation–optimization; ABS = agent-based simulation; AC = artificial case; BSS = bike sharing system; na = not applicable.

There are many works that study the design and operation of a BSS. The optimization models developed in each study differ in features, such as the level of service or the costs of the system, that are considers in their formulation. These features are expressed in the objective function and the type of constraints of the models. Most of the reported research includes constraints related to various costs of a BSS or their objective function refers to the cost or profit of the system. This means that the level of service offered by the BSSs designed by these optimization models is limited by the available budget. It is also observed that the optimization models concern the design and operation of either free-floating systems or docked systems, whose characteristics differ. The main difference is that the design of the docked system requires the installation of stations, while in the free-floating system there may be no stations. Moreover, it is observed that only one study (*39*) approaches the design of a mixed-fleet bike and e-bike BSS. Therefore, there is no study that simultaneously designs a BSS consisting of a mixed-fleet—bike and e-bike—where the bike system is free floating, while the e-bike system is docked. Finally, none of the reviewed studies consider extreme situations and disturbances in the PTS, such as a pandemic situation and distancing constraints. In the case that the system costs are not considered, it leads to the development of an optimization model that can provide the design and operation of a BSS designed to provide increased mobility capacity. In addition, a mixed-fleet BSS can serve different cases of people, such as young people, the elderly, or people with vulnerable health conditions and those traveling different distances. A hybrid system can cope with the increased demand that results from the distancing constraints, as it combines the positives of docked and free-floating systems. To the best of the authors’ knowledge, this is the first study that considers a pandemic situation and mobility needs arising because of distancing constraints on the PTS and seeks to integrate the PTS and the BSS with regard to mobility capacity. That is, the study deals with the creation of a resilient PTS that can provide mobility capacity in extreme and special situations. In addition, it is the first research to develop a BSS optimization model that incorporates the design and operation of a mixed-fleet system as well as a different design approach—free floating and docked—of the two-mode system. All the above features create an advanced optimization model that optimizes the design and operation of bike and e-bike systems separately but simultaneously.

## Modeling Approach

In this section, we introduce the applied modeling framework, the data analysis approach to integrate the PTSs and BSSs, and the developed optimization model.

### Modeling Framework

**Figure 2.**
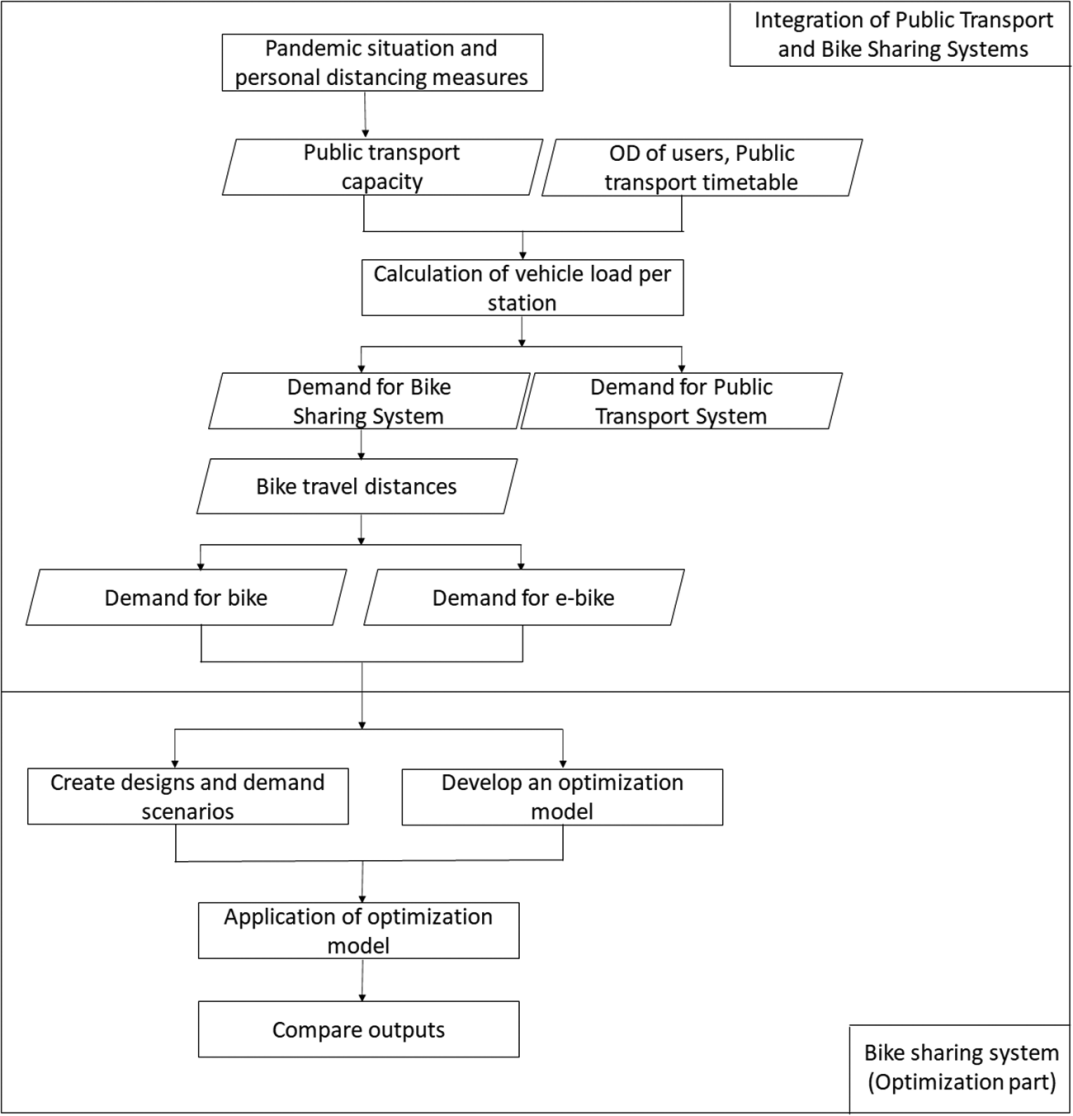
Modeling framework, showing the various steps of the process and their sequence. The rectangles represent a process or a state, while parallelograms are used for input or output operation. The arrows connect the symbols and indicate the flow of the process and information.

The modeling framework of the study, which includes the integration of the PTSs and BSSs and the optimization model of the MFHBSS under the impacts of social distancing measures, is shown in Figure 2.

### Integration of Public Transport and Bike Sharing Systems

The objective of the integration of the two systems is to find the demand per system so as to then adjust the capacities in an integrated fashion. The approach to achieving this integration is based on the factors of the pandemic, namely the capacity constraints on the PTS and the new bike system network. The first step is to create a mathematical data analysis model that calculates the permissible boarding of demand per station of each pubic transport vehicle and exports the unsatisfied demand per station. The model gives priority to boarding passengers with the farthest destination. The inputs of the model are the capacity of the vehicle (with regard to passengers that can be transported by the vehicle), the percentage of permissible occupancy (i.e., the allowed load of passengers) because of the distancing constraints, the number of schedules, and the demand of the PTS. The outputs of the model are the vehicle load, the demand satisfied by the vehicle and its destination station, and the unsatisfied demand and the respective destination station. Therefore, the destination pairs of the unsatisfied demand are known. The result of this model is the distribution of demand in PTSs and BSSs.

Here, P is the set of stations indexed by i and j, k is the index for the schedule, $ld_{k,i}$ is the load of schedule k in station i, $\mathrm{de}m_{k,i,j}$ is the demand from station i to station j for schedule k, $\mathrm{unde}m_{k,i,j}$ is the unmet demand from station i to station j for schedule k, ac is the allowed capacity on the vehicle, $ub_{k,i}$ is the debarkation passengers in station i for schedule k, and $b_{k,j}$ is the boarding passengers at station i for schedule k.

Subsequently, the integration approach is described. For the first station 1 of the line, in the case in which the vehicle demand is lower than the available vehicle capacity because of the distancing constraints, the following holds:

(1) $$ld_{k,1}=\sum_{j\in P}(\mathrm{de}m_{k,1,i})$$

(2) $$\mathrm{unde}m_{k,1,j}=0\;\;\forall j\in P$$

Equation 1 states that the load of the vehicle schedule k at the first station is equal to the sum of the demand of the first station to all the other stations of this line. The unsatisfied demand of the schedule k from the first station to any other station is zero (Equation 2).

In the case in which the demand is higher than the available vehicle capacity because of the distancing constraints, the following holds:

(3) $$ld_{k,1}=\mathrm{ac}$$

(4) $$\mathrm{unde}m_{k,1,j}=\sum_{j\in P}(\mathrm{de}m_{k,1,j})\;-\mathrm{ac}\;\;\forall j\in P$$

The load of the schedule k at the first station is equal to the allowed capacity of the vehicle because of the distancing constraints (Equation 3). In this case the unsatisfied demand, Equation 4, of the schedule k from the first station to a station j is equal to the sum of the demand from the first station to all other stations minus the allowed capacity on the vehicle.

For all other stations of the line, in the case in which the vehicle load is lower than the available vehicle capacity because of the distancing constraints, the following holds:

(5) $$ub_{k,i}=\sum (\mathrm{de}m_{k,1:i,i}-\mathrm{unde}m_{k,1:i,i})$$

(6) $$b_{k,i}=\sum (\mathrm{de}m_{k,i,i+1:P})$$

(7) $$ld_{k,i}=ld_{k,i-1}-ub_{k,i}+b_{k,i}$$

(8) $$\mathrm{unde}m_{k,i,j}=0\;\;\forall j\in P$$

Equation 5 determines that the passengers who disembark from the schedule k in station i are equal to the total demand of all the previous stations that have as a destination the station i if you exclude the unsatisfied demand of all the previous stations that have as a destination the station i, while passengers boarding the schedule k at the station i are equal to the total demand from the station i to all subsequent stations (Equation 6). The load of the schedule *k* at the station i is equal to the load of the schedule k at the previous station (i−1) and the passengers who want to board at station i minus the passengers who want to disembark at the station i (Equation 7). Equation 8 states that there is no unsatisfied demand for the schedule k from station i to any other station j.

In the case in which the vehicle load is higher than the available vehicle capacity because of the distancing constraints:

(9) $$ld_{k,i}=\mathrm{ac}$$

(10) $$\mathrm{unde}m_{k,i,j}=ld_{k,i-1}-\mathrm{ac}-ub_{k,i}+\mathrm{de}m_{k,i,j}\;\;\forall j\in P$$

Equation 9 specifies that the load of schedule k at station i is equal to the allowed capacity of the vehicle, while the unsatisfied demand of schedule k from station i to station j is equal to the vehicle load at the previous station (i−1) and the demand of station i to the station j after subtracting the allowed capacity of the vehicle and passengers disembarking at station i (Equation 10).

The second step of the integration approach is to separate BSS demand into bike demand and e-bike demand. This can be achieved based on the travel distances. The data for this step are the unsatisfied demand from the PTS, the travel distances of the bike network, which has been extended because of the pandemic situation, between the stations of the PTS with unsatisfied demand, and the rates of use per mode—bike and e-bike—for specific distance clusters. The result of this integration is the separation of the existing demand of the PTS into the demand of the PTS and the demand for bikes and the demand for e-bikes of the BSS.

### Optimization Model

We introduce an optimization model to determine the optimal design and operation of a hybrid mixed-fleet BSS compensating for the limited capacity in the PTS because of social distancing constraints. The notation used to represent the elements of the optimization model is shown in Table 2.

**Table 2.** Optimization Model Notation

Sets
*J*: set of stations, with indices *i* and *j*
*T*: set of time periods, with index *t*, *T* = 1,…, *t*
P⊆T: set of time periods, with index *t*, *P* = 2,…, *t*
Decision variables
$y_{i}$: is 1 if the bikes station is opened and 0 otherwise
$x_{i,j,t}$: proportion of covered bikes demand from station *i* to station *j* in period *t*
$r_{i,j,t}$: number of bikes relocated from *i* to *j* at period *t*
$\upsilon_{i,t}$: number of bikes in station *i* at the beginning of period *t*
$Tu_{t}$: total bike fleet size of the system
$h_{i}$: is 1 if the e-bike station is opened and 0 otherwise
$v_{i}$: number of e-bike docks in station *i*
$w_{i,j,t}$: proportion of covered e-bike demand from station *i* to station *j* in period *t*
$s_{i,j,t}$: number of e-bikes relocated from *i* to *j* at period *t*
$b_{i,t}$: number of e-bikes in station *i* at the beginning of period *t*
$Te_{t}$: total e-bike fleet size of the system
$u_{i,j,t}$: bike demand from *i* to *j* in period *t*
Parameters
$e_{i,j,t}$: e-bike demand from *i* to *j* in period *t*
$z_{\max}$: maximum available bikes in a station
$v_{\min}$: minimum capacity of e-bike station
$v_{\max}$: maximum capacity of e-bike station
$p_{\min}$: minimum percentage of used capacity in an e-bike station *i* at the beginning of period *t*
$p_{\max}$: maximum percentage of used capacity in an e-bike station *i* at the beginning of period *t*

This is achieved by maximizing the covered demand considering location and relocation constraints. Most bike sharing models assume a cost-based optimization approach; in this case, however, we set out to examine how many stations with how many bikes would be needed to cover the excess demand for public transit. Therefore, we model the problem based on a maximal covering location approach, introduced by Church and ReVelle (*20*), which maximizes the demand covered by the BSS.

The model has some inputs and outputs. The inputs are a set of stations, the demand of the bike and e-bike systems, the values for the parameters of maximum and minimum capacity, the maximum available bikes in a virtual station, the maximum and minimum percentage of used capacity of the e-bike system, and the number of time periods. A virtual station is a station that does not exist yet but may be opened to cover the additional demand (as a result of the decisions proposed by the optimization model). That is, within the model, all stations are treated as virtual stations. For simplicity, we subsequently refer to virtual stations only as stations. Furthermore, we focus on the capacity and supply of a system. This is an important assumption, since we want to provide a viable alternative to PTS capacity and we must ensure that most PTS users would be able to use this alternative, even if they are not in physical shape for longer (non-electric) bike rides. That is, we aim to satisfy a certain share of e-bike demand, even if users may switch between modes if they cannot use their preferred choice. Time periods are essentially the number of periods into which a day is divided. This number can be determined in each case study based on its data. The model satisfies the demand of the system but also relocates bikes and e-bikes, so there should be a balance between them when determining the number of time periods. In addition, the values of maximum and minimum capacity and percentage of used capacity can be determined based on the literature, or there can be variation in their range of values. This depends on the requirements of each case study. The parameter for the maximum number of bikes in a station depends on each case of study, that is, the availability of public space. The outputs of the optimization model are the covered demand of the hybrid mixed-fleet BSS, the number of stations, the sizes of the bike and e-bike fleets, the number of bikes and e-bikes at stations in each time period, the number of relocated bikes and e-bikes per station pair in each time period, the portion of covered demand per station pair in each time period, and the number of stations of the e-bike system.

In the following, the model is presented:

(11) $$\begin{array}{c} \mathrm{Max}\;Z=\sum_{i\in J}\;\sum_{j\in J}\;\sum_{t\in T}(u_{i,j,t}*x_{i,j,t})\\ \;\;\;\;\;\;\;\;\;\;\;+\sum_{i\in J}\;\sum_{j\in J}\;\sum_{t\in T}(e_{i,j,t}*w_{i,j,t}) \end{array}$$

subject to:

(12) $$\begin{array}{c} \;\;\;\;\;\;\;\;\;\;\;\;\;\;\upsilon_{i,t}=\upsilon_{i,(t-1)}-\sum_{j\in J}u_{i,j,(t-1)}*x_{i,j,(t-1)}\\ +\sum_{j\in J}u_{j,i,(t-1)}*x_{j,i,(t-1)}+\sum_{j\in J}r_{j,i,(t-1)}-\sum_{j\in J}r_{i,j,(t-1)}\\ \;\;\;\;\;\;\;\;\;\;\;\;\;\;\;\forall i,j\in J,t\in T \end{array}$$

(13) $$\begin{array}{c} b_{i,t}=b_{i,(t-1)}-\sum_{j\in J}e_{i,j,(t-1)}*w_{i,j,(t-1)}\\ +\sum_{j\in J}e_{j,i,(t-1)}*w_{j,i,(t-1)}+\sum_{j\in J}s_{j,i,(t-1)}-\sum_{j\in J}s_{i,j,(t-1)}\\ \;\forall i,j\in J,t\in T \end{array}$$

(14) $$u_{i,1}=u_{i,T}\;\;\forall i\in J$$

(15) $$b_{i,1}=b_{i,T}\;\;\forall i\in J$$

(16) $$v_{i}\leq v_{\max}*h_{i}\;\;\forall i\in J$$

(17) $$v_{i}\geq v_{\min}*h_{i}\;\;\forall i\in J$$

(18) $$\upsilon_{i,t}\geq \sum_{j\in J}(u_{i,j,t}*x_{i,j,t})\;\;\forall i,j\in J,t\in T$$

(19) $$b_{i,t}\geq \sum_{j\in J}(e_{i,j,t}*w_{i,j,t})\;\;\forall i,j\in J,t\in T$$

(20) $$b_{i,t}\leq p_{\max}*v_{i}\;\;\forall i\in J,t\in T$$

(21) $$b_{i,t}\geq p_{\min}*v_{i}\;\;\forall i\in J,t\in T$$

(22) $$\upsilon_{i,t}\leq z_{\max}*y_{i}\;\;\forall i\in J,t\in T$$

(23) $$\sum_{j\in J}r_{i,j,t}\leq \upsilon_{i,t}\;\;\forall i\in J,t\in T$$

(24) $$\sum_{j\in J}s_{i,j,t}\leq \;b_{i,t}\;\;\forall i\in J,t\in T$$

(25) $$Tu_{t}=\sum_{i\in J}\upsilon_{i,t}\;\;\forall t\in T$$

(26) $$Te_{t}=\sum_{i\in J}\;b_{i,t}\;\;\forall t\in T$$

(27) $$x_{i,j,t}\leq 1\;\;\forall i,j\in J,t\in T$$

(28) $$w_{i,j,t}\leq 1\;\;\forall i,j\in J,t\in T$$

(29) $$w_{i,j,t}\leq h_{i}\;\;\forall i,j\in J,t\in T$$

(30) $$w_{i,j,t}\leq h_{j}\;\;\forall i,j\in J,t\in T$$

(31) $$x_{i,j,t}\leq y_{i}\;\;\forall i,j\in J,t\in T$$

(32) $$x_{i,j,t}\leq y_{j}\;\;\forall i,j\in J,t\in T$$

(33) $$r_{i,j,t}\geq 0\;\;\forall i,j\in J,t\in T$$

(34) $$s_{i,j,t}\geq 0\;\;\forall i,j\in J,t\in T$$

(35) $$x_{i,j,t}\geq 0\;\;\forall i,j\in J,t\in T$$

(36) $$w_{i,j,t}\geq 0\;\;\forall i,j\in J,t\in T$$

(37) $$h_{i}\;\in \{0,1\}\;\;\forall i\in J$$

(38) $$\upsilon_{i,t},b_{i,t},v_{i},r_{i,\mathrm{jt}},s_{i,j,t},Tu_{t},Te_{t}\;\;\in N\;\;\forall i,j\in J,t\in T$$

The objective function 11 of this mixed-integer linear programming (MILP) model consists of two terms. The first term is the covered demand for conventional bikes, while the second term is the covered demand for e-bikes. The objective function maximizes the covered demand by the BSS. Constraint 12 determines the available bikes at station i at period t. The first term of the constraint refers to the number of available bikes at station i in the previous period. The second and third terms refer to the number of bikes that left or arrived at the station i, respectively, in the previous period, while the fourth and fifth terms refer to the bikes transported to or from the station i, respectively, at the previous period. Constraint 13 determines the number of available e-bikes at station i at period t. Constraints 14 and 15 state that the bike and e-bike fleet of the system remains the same between the first and the last period. The capacity of an e-bike station is limited by Constraints 16 and 17. Constraint 16 specifies the upper capacity limit (number of docks), while Constraint 17 specifies the lower capacity limit. The available bikes at the station i should meet the demand of the station (Constraint 18), and the available e-bikes at the station i should meet the demand of the station (Constraint 19). Stations should always have available e-bikes as well as available docks for parking. This is achieved by Constraints 20 and 21. Constraint 20 specifies that the available e-bikes at the station i at the period t should not exceed a specific number, and there should be a minimum number of e-bikes at the station (Constraint 21). Constraint 22 sets a limit on the maximum number of available bikes at a station. The relocated bikes from the station i at the period *t* should not exceed the available bikes at the station i at that period (Constraint 23). Decision variable $y_{i}$ indicates whether a new station should be opened in location i. The corresponding constraint for the e-bike system is Constraint 24. Constraints 25 and 26 specify the total bike and e-bike fleet of the BSS, respectively. The portion of covered demand from stations i to j at the period t cannot exceed the value 1 (Constraint 27). The corresponding constraint for the e-bike system is Constraint 28. The demand for the bike and e-bike system can only be served by existing (virtual) stations (Constraints 29–32). Constraints 33–38 specify the domain of the decision variables.

## Case Study and Numerical Results

In this section, we present the underlying case study, the considered scenarios, and the obtained computational results.

### Case Study

In this study, the area of investigation is the city center of Milan and the studied systems are the subway system and the public BSS. Milan is located in northern Italy and is the capital of the administrative region of Lombardy. The Milan subway has four lines (M1, M2, M3, and M5) and 106 stations. The public BSS started operating at the end of 2008. At present, the system has 4280 bikes and 1150 e-bikes. The number of operational stations is 320. Subway system demand data, that is, the origin–destination data, is generated based on higher-level data from the subway system in Milan. Firstly, we use information about the subway system and its demand. The available information is related to the daily passenger demand per line in 2018, the total daily system demand for 2019, and the passenger use of each station (low, medium, or high) during the day at time intervals of half an hour for the first week of April 2021, and the system’s peak hours. Based on this data, the generation of the origin–destination pairs is performed separately for each subway line.

### Scenarios and Designs

There are three demand scenarios (SClow, SChigh, SClockdown). SClow and SChigh consist of the unsatisfied demand of the PTS and the demand of different days of the public BSS (4 and 8 April 2019, respectively), while SClockdown consists of the demand of the BSS on 8 April 2020. The three different demand scenarios are used as inputs for the designs. The basic demand scenario that most designs consider is SClow. SChigh and SClockdown will be used as inputs for a few designs. SClow is the BSS combination of the unsatisfied demand for the PTS (bike demand: 44,700 and e-bike demand: 12,700) and the demand for the BSS on 4 April 2019 (bike demand: 800 and e-bike demand: 200). In the SClockdown scenario, the demand for the BSS is based on 8 April 2020 (bike demand: 150 and e-bike demand: 90). The BSS during the period of strict lockdown is in low demand on all the investigated days. The date 8 April 2020 is chosen because it is one of the most demanding days with regard to demand for the system during the lockdown period. SClockdown does not include unsatisfied demand from the PTS, as human movements were very low because of the strict lockdown. SChigh includes the unsatisfied demand for the PTS (bike demand: 44,800 and e-bike demand: 12,700) and an increased demand for the BSS on 8 April 2019 (bike demand: 3500 and e-bike demand: 550). All demand numbers are rounded to indicate that they are estimates.

**Figure 3.**
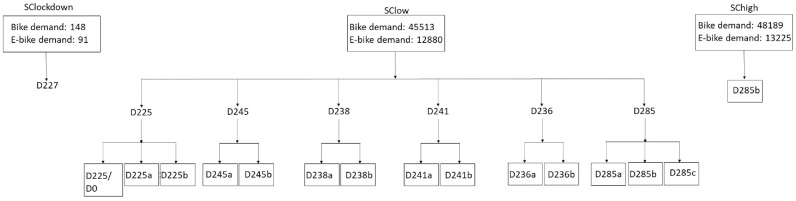
Developed scenarios and designs for model application. There are three demand scenarios and 15 designs in which their parameters are differentiated.

The designs are created based on the needs of the BSS. The parameters that differ in the designs are the number and the location of (virtual) stations in the network, the maximum number of bikes per station, and the capacity (number of docks) of the e-bike stations. The first categorization of the designs concerns the number and the location of stations and e-stations. Based on these two parameters, seven basic designs are created. Each design is named with the capital letter D from the word design and the number of stations. These are D225, D245, D238, D241, D236, D285, and D227. We have limited the scenarios to this relatively narrow band based on an initial analysis that gave us lower bounds based on the required capacity and upper bounds based on an estimation of available space in the considered area. The locations of the new stations are close to subway stops. Then, the other two parameters are considered. Two main types of designs emerge from this separation, Da and Db. Da has a maximum number of bikes per station at 50 bikes, a minimum number of e-bikes docks at 10, and a maximum number of e-bike docks at 25, while Mb has 80 bikes, 10 e-bikes docks, and 40 e-bikes docks. Design D0 is the design of the BSS in 2019 with parameter values of 20, 1, and 10, respectively. The specifications of design D0 also apply to D227. The final design that is created is the Mc, in which there is no limit to the maximum number of bikes, while the maximum and the minimum number of e-bike docks are 10 and 200, respectively. The common features of all designs are the following. The system is studied for 6 h, 15:00–21:00. The optimization model requires the definition of time periods. This period is selected because it includes the evening peak hour. The choice of the evening peak hour versus the morning peak hour is based on the BSS. The BSS was in greater demand during the evening peak hour in 2019. In this case, there are three time periods, t1: 15:00–17:00; t2: 17:00–19:00; and t3: 19:00–21:00. Therefore, the demand for the system is divided into these three time periods. In addition, the maximum and minimum used capacity percentages on e-stations are 25% and 75%, respectively. Figure 3 shows the scenarios and designs.

### Experimental Results

The first analysis is related to the unsatisfied demand of the PTS. Unsatisfied demand arises from the use of the mathematical model for the integration of the two systems—PTS and BSS—and the demand of the PTS. Demand for the PTS is considered hourly. It is therefore divided equally among the schedules operated on each subway line per hour. For the different demand scenarios (SClow, SChigh, SClockdown), different percentages of unsatisfied PTS demand caused by social distancing constraints are considered. For the SClow and SChigh scenarios, a value of 30% is assumed, while the SClockdown scenario considers 0% because of generally low public transit demand in this scenario. Then, the unsatisfied demand is divided into bike and e-bike demand based on the bike network travel distances and the rates of bike and e-bike use for specific travel distance intervals. About 22% of the total demand resulting from the integration of the two systems is the demand for e-bikes and 78% is the demand for bikes.

The analysis of the BSS considering aspects of the pandemic situation uses the optimization model for a MFHBSS and the various designs developed. The outputs of the model are the number of stations, the covered demand and the size of the fleets, and the relocation. Table 3 shows the outputs of some designs, for which the correlations are analyzed below. The system demand is the same for all designs presented in Table 3.

**Table 3.** Inputs and Outputs of Some of the Developed Designs

	Inputs			
	D0	D285a	D285b	D285c
Number of stations	225	285	285	285
Maximum number of bikes	20	50	80	Unlimited
Maximum number of docks	10	25	40	200
	Outputs			
	D0	D285a	D285b	D285c
Number of selected stations	169	211	215	210
Number of stations	225	285	285	285
Covered bike demand	2732	6599	9685	45,513
Covered e-bike demand	871	2160	3279	8896
Bike fleet	1213	3105	4488	30,959
E-bike fleet	627	3267	4886	20,445
Relocated bikes	1146	2596	3746	30,329
Relocated e-bikes	474	2045	3865	13,019

**Figure 4.**
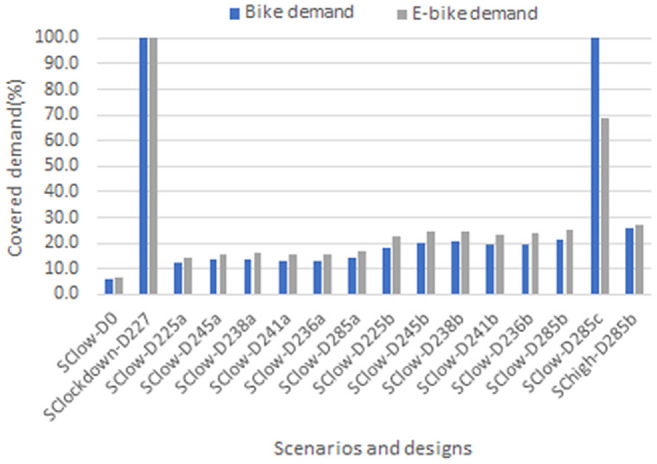
Covered demand per design. For each design, the demand for the bike system is presented in blue, while the demand for the e-bike system is presented in gray. (Color online only.)

Initially, the covered demand per scenario is analyzed. Covered demand in design D0 is just 6% for the bike system and just under 7% for the e-bike system. In all other designs, there is at least a doubling of the covered demand rates (2.1–2.4 times). Only D227 fully meets the demand of both systems, which is logically because of the low demand of the input SClockdown. The other design that has a full coverage of bike system demand and high coverage of e-bike system demand is D285c. The high coverage rates, in this case, are related to the design features of the system, that is, an unlimited number of bikes per station and the large capacity of the e-stations. In all other designs, it is observed that the covered demand is higher in percentage for the e-bike system. This may be because of the lower demand requirements for this system. In addition, it is observed that the Da designs, which have lower values in the capacity of their stations, have lower covered demand compared to the scenarios in Db, which are characterized by higher station capacity. Demand rates in designs Db show a steady growth rate compared to the corresponding Da designs. Figure 4 gives an overview of the analyzed scenarios and designs. It becomes clear that there are only minor differences in covered demand between the high and low demand scenarios. This can be explained, because only the BSS demand changes in these scenarios and the BSS demand is significantly lower than the unsatisfied PTS demand.

Considering the relation between the covered demand and the fleet size, the general trend in the bike system is that the covered demand increases with the increase of the bike fleet. It is also observed that in each design the fleet size is about half in relation to the covered demand. This observation does not apply only to the D227 design in which the fleet size and the covered demand are almost the same and the D285c design in which the fleet size is lower than the covered demand but not to the trend prevailing in the other designs. The e-bike system does not show the same trends as the covered demand and the fleet size as the bike system. Designs D227 (low demand) and D285c (high station capacity specifications) have a large fleet size in relation to covered demand for both systems. This indicates that the BSS based on its design has service specifications, such as the availability of bikes and e-bikes, regardless of the size of its demand.

The analysis of covered demand and the number of stations is performed separately for the two systems (see Figure 4). With regard to design D227, the demand of both systems is fully covered. Although the demand is low, the station network is relatively large (227 stations and 107 e-stations). This indicates that demand is spread across the study area and wide station coverage is needed even in this case. For both systems, it is observed that the increase of the stations is not in line with the increase of the covered demand in some cases. In cases where the number of selected stations is the same or almost the same, designs with high-capacity specifications (Db designs) satisfy more demand. For the bike system, in the case of the scenario with higher demand, it is observed that the same number of stations can satisfy more demand.

**Figure 5.**
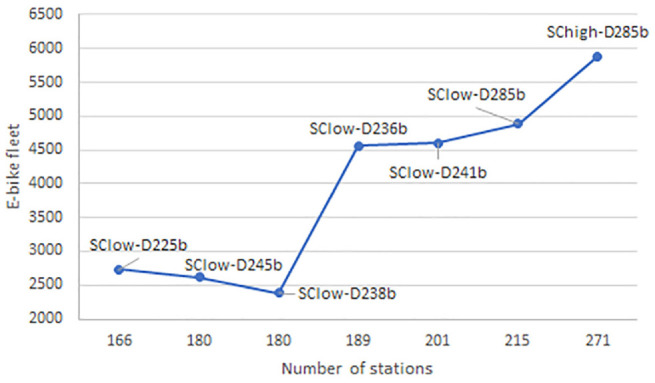
For each design, the relationship between the number of stations (*x*-axis) and the size of the e-bike fleet (*y*-axis) is presented. *Note*: OD = origin–destination.

**Figure 6.**
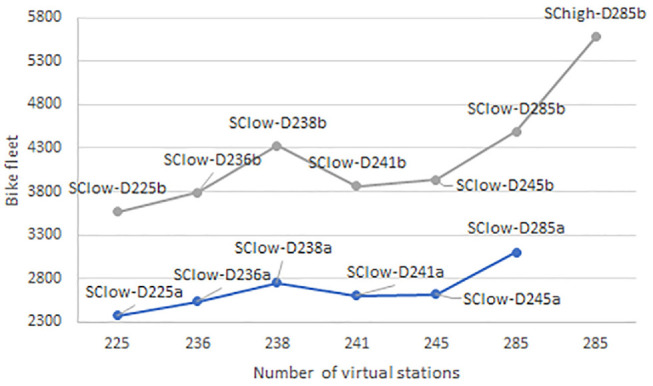
For each design, the relationship between the number of stations (*x*-axis) and the size of the bike fleet (*y*-axis) is presented. The gray color illustrates the Db designs, while the blue color illustrates the Da designs. (Color online only.)

An interesting analysis is the number of stations compared to the size of the fleet (see Figures 5 and 6). There can be no clear trend for the Da designs of the e-bike system. For the Db designs, it is observed that the fleet presents slightly differently for the same number of stations (180). However, the design with the lowest fleet size satisfies higher demand. In addition, the difference in the fleet size between a system with 180 stations and a system with 189 stations is significant. However, this does not mean an increase in covered demand. In other cases, as the number of stations increases, so does the fleet size as well as the covered demand.

The bike system presents uniformity between the results of designs with low (Da) and high (Db) capacity specifications. The size of the fleet increases as the number of stations increases. This statement differs only when the number of network stations is 241 or 245. In these two cases, it is observed that the bike fleet shows a decrease. However, this is in line with the demand coverage. In the case in which the demand for the system increases, the same number of stations satisfies more demand (design D285b under different demand scenarios).

The size of the bike relocation follows an upward trend as the size of the bike fleet increases (see Table 4). In a few cases, there is a decrease in the size of bike relocation, while there is an increase in the size of the fleet. The size of the relocation is always smaller than the size of the fleet. Only the case of design D227 is an exception. This may be because there are many stations (227 stations) relative to the low fleet size (137 bikes). It should also be noted that there is no difference in results between designs with low-capacity (Da) and high-capacity (Db) specifications on stations. The e-bike system cannot be characterized by stability in the relation between the fleet and relocation size. The size of the fleet is higher than the size of the relocation for all designs beyond one design. In the designs with stations of high-capacity specifications, there is more relocation in relation to the size of the fleet than in the designs with low-capacity specifications.

**Table 4.** The Bike Fleet and the Relocation Sizes per Design

Designs	Bike fleet size	Relocation size	E-bike fleet	Relocation size
SClockdown-D227	137	178	402	373
SClow-D0	1213	1146	627	474
SClow-D225a	2379	1866	1192	1500
SClow-D236a	2542	2200	1927	1147
SClow-D241a	2608	2197	2610	1936
SClow-D245a	2622	2146	1874	1387
SClow-D238a	2748	2287	2769	2257
SClow-D285a	3105	2596	3267	2045
SClow-D225b	3568	3024	2729	2142
SClow-D236b	3786	3264	4564	4438
SClow-D241b	3866	3508	4599	4506
SClow-D245b	3939	3269	2614	2030
SClow-D238b	4325	3907	2382	2130
SClow-D285b	4488	3746	4886	3865
SC2-D285b	5575	5180	5884	4880
SClow-D285c	30,959	30,329	20,445	13,019

The final analysis concerns the system costs. The total cost consists of the purchase costs and the relocation costs. The cost of buying e-bikes is the highest cost. In most cases, low station capacity designs (Da) have lower final costs than higher station capacity designs (Db). The cost of relocating (e)-bikes is relatively low compared to the purchase costs of the fleet. For both systems, an increase in the fleet size does not imply an increase in relocation costs. Finally, it should be noted that there is no correlation between costs and covered demand.

## Conclusions and Future Work

During the COVID-19 pandemic, many sectors have been affected by the government measures to reduce the spread of the virus. The transport sector is one of the sectors most affected by these measures. The mobility capacity of PTSs has been reduced by the implementation of the distancing measures. That is, part of the system’s capacity has no longer been provided. This leads to the need of finding new forms of PTSs that can offer mobility capacity to those who need it during the pandemic or similar future situations. The integration of bike sharing and existing PTSs is one form of creating such new systems. In this work, we have aimed to design a BSS to meet the extraordinary needs for mobility during a pandemic situation. The first need that arises is to provide a solution that not only serves all groups of people, that is, young and elderly, but also considers different trip distances. This need can be met by using a mixed fleet, that is, bikes and e-bikes. Conventional bikes may be preferred for shorter distances and by people in better physical condition, while e-bikes may be preferred by people with health conditions and, generally, for longer distances since their use does not cause much physical fatigue. The second need that likely arises in a pandemic scenario is the increased demand for transportation because of the reduced mobility capacity of PTSs caused by the distancing measures. A BSS with a free-floating bike system and docked e-bike system addresses this need and increases the mobility capacity in the BSS.

To achieve the aforementioned integration, a data analysis model that integrates the demand needs of PTSs and BSSs has been developed. Moreover, a model for optimizing a MFHBSS has been proposed. The optimization model is used to evaluate different designs and demand scenarios to identify the prevailing trends for the design and operation of a MFHBSS that aims to provide mobility capacity during a pandemic (and similar disruptive events). The city of Milan is used as a case study for the implementation of the approach. In this way, this work overcomes a research gap in providing mobility capacity during a pandemic (or similar capacity-limiting events) by integrating PTSs and BSSs and adds to earlier work on the interplay of BSSs and PTSs as presented in case studies in different cities (*15*[Bibr bibr16-03611981221123241]–*17*).

The main findings and policy implications are summarized subsequently.

- Based on our analysis, we observe that 30% of the demand for the evening peak hour of the subway system in Milan cannot be satisfied because of distancing measures. In an effort to maintain mobility capacity, we would propose the integration of the BSS with the PTS. Therefore, we recommend cooperation between the operators of the PTSs and BSSs. This could be achieved by means of an integrated planning system that adjusts the PTS capacities (e.g., with regard to train frequencies) and BSS capacities (e.g., with regard to new stations, bicycles, or relocations) according to predicted real-time demands. For the customer, an integrated app would act as a “single window” and provide instructions as well as assignments, routing, and fare information.
- The current BSS in Milan can only compensate for 6% of the PTS and its own demand. Our advice to BSS operators to increase this percentage is a system design with different types of bikes, that is, the creation of e-bike stations and free-floating bike systems. The PTSs may increase frequencies on their lines or spread out demand across the day. In addition, PTSs could require 100% compliance with face mask rules (if not done already) and investigate the effectiveness of their heating, ventilation, and air conditioning systems.
- The dual strategy with a free-floating bike system and a docked e-bike system and the creation of bike stations near the subway stations with unsatisfied demand increases the covered demand at least twice (2.1–2.4 times). We therefore propose that the BSS providers should invest in the construction of stations near subway stations.
- An increase of the capacity of the e-stations and the available bikes in stations by about 37% results in an additional increase of the covered demand by 6.5%–7.5%. Based on this result, we recommend that BSS operators pay special attention to the capacity specifications of the stations during their design.
- As far as the free-floating bike system is concerned, it is also observed that there is stability in the ratio of the covered demand and bike fleet. The ratio (covered demand/fleet size) is between 2.13 and 2.36. It is suggested that the Milan BSS takes this into account for a rough initial fleet forecast. In this way, it will provide the necessary mobility but will also be a careful investment.
- The demand for the e-bike system is 22% of the unsatisfied demand and 78% for the conventional bike system. Based on this insight and the instability of the e-bike system results, it is recommended that BSS operators should carefully invest in the e-bike system and then extend it based on needs that arise.
- In our results, we observe that the system fully satisfies its low demand during the lockdown period. However, the fleet needs in relation to the covered demand are high. The covered demand to fleet size ratio is 1.1 for the bike system and 0.23 for the e-bike system. Also, the network of stations is wide, with 107 e-stations and 227 stations. This shows that the system has a spatial range of demand. We would therefore advise BSS operators to develop a wide network of stations.
- The results show differences in fleet needs, 137–5575 bikes and 402–5884 e-bikes, and station needs, 107–271 e-stations and 225–285 stations. We would therefore also advise BSS operators to install some stations on mobile trailers that can be easily moved. An additional piece of advice would be that the available fleet on the system should be period-based.
- To fully meet the bike system demand, 30,959 conventional bikes are needed, while 20,445 e-bikes are needed for 70% coverage of e-bike demand. In addition, there is no limit to the available bikes per station, while the maximum number of docks per station is 200. It is concluded that the BSS cannot fully counterbalance for the limited capacity of the PTS.
- The BSS may have been designed based on the needs of the pandemic, but the use of such a system may be more extensive. The basic criterion for the implementation of a MFHBSS is its ability to satisfy the mobility needs of each case.

Nevertheless, this work also has some limitations that could be addressed in future research. The approach of the system integration can be done based on the travel time and be more pandemic-oriented with the integration of an application that detects the movement of infected people. Therefore, the user will be informed in real-time about the chances of meeting an infected person and will choose accordingly the transport means they desire. It should furthermore be noticed that the PTS in Milan involves a fairly extensive tram, trolley-tram, and bus network, in addition to its four subway lines. In a pandemic situation, these other PTS modes may also have to deal with reduced capacities. Future research could also take into account this unmet demand to investigate the impact of other modes of public transportation on BSSs and other shared mobility systems. Moreover, the formulation of the developed optimization model refers to the design and operation of a docked e-BSS. It is suggested that future research also includes the choice of whether to use an e-bike based on its battery level and/or consider the rebalancing processes.
